# Automated Hypofractionated IMRT treatment planning for early-stage breast Cancer

**DOI:** 10.1186/s13014-020-1468-9

**Published:** 2020-03-17

**Authors:** Ting-Chun Lin, Chih-Yuan Lin, Kai-Chiun Li, Jin-Huei Ji, Ji-An Liang, An-Cheng Shiau, Liang-Chih Liu, Ti-Hao Wang

**Affiliations:** 1 Department of Radiation Oncology, China Medical University Hospital, China Medical University, Taichung, Taiwan; 2 Department of Medicine, China Medical University, Taichung, Taiwan; 3 Department of Biomedical Imaging and Radiological Sciences, National Yang-Ming University, Taipei, Taiwan; 4 Department of Biomedical Imaging and Radiological Science, China Medical University, Taichung, Taiwan; 5 Department of Surgery, China Medical University Hospital, Taichung, Taiwan

**Keywords:** Automation, Treatment planning, Autoplanning, Hypofractionation, IMRT, Early-stage, Whole-breast irradiation, Left-sided breast cancer

## Abstract

**Background:**

Hypofractionated whole-breast irradiation is a standard adjuvant therapy for early-stage breast cancer. This study evaluates the plan quality and efficacy of an in-house-developed automated radiotherapy treatment planning algorithm for hypofractionated whole-breast radiotherapy.

**Methods:**

A cohort of 99 node-negative left-sided breast cancer patients completed hypofractionated whole-breast irradiation with six-field IMRT for 42.56 Gy in 16 daily fractions from year 2016 to 2018 at a tertiary center were re-planned with an in-house-developed algorithm. The automated plan-generating C#-based program is developed in a Varian ESAPI research mode. The dose-volume histogram (DVH) and other dosimetric parameters of the automated and manual plans were directly compared.

**Results:**

The average time for generating an autoplan was 5 to 6 min, while the manual planning time ranged from 1 to 1.5 h. There was only a small difference in both the gantry angles and the collimator angles between the autoplans and the manual plans (ranging from 2.2 to 5.3 degrees). Autoplans and manual plans performed similarly well in hotspot volume and PTV coverage, with the autoplans performing slightly better in the ipsilateral-lung-sparing dose parameters but were inferior in contralateral-breast-sparing. The autoplan dosimetric quality did not vary with different breast sizes, but for manual plans, there was worse ipsilateral-lung-sparing (V_4Gy_) in larger or medium-sized breasts than in smaller breasts. Autoplans were generally superior than manual plans in CI (1.24 ± 0.06 vs. 1.30 ± 0.09, *p* < 0.01) and MU (1010 ± 46 vs. 1205 ± 187, *p* < 0.01).

**Conclusions:**

Our study presents a well-designed standardized fully automated planning algorithm for optimized whole-breast radiotherapy treatment plan generation. A large cohort of 99 patients were re-planned and retrospectively analyzed. The automated plans demonstrated similar or even better dosimetric quality and efficacy in comparison with the manual plans. Our result suggested that the autoplanning algorithm has great clinical applicability potential.

## Background

Breast cancer is the second most prevalent cancer and the second leading cause of cancer death among women worldwide, with a slight but stable increase in its incidence in the most recent decade [1]. At our center, breast cancer patients account for approximately one-third of all patients requiring curative radiotherapy. Fortunately, most patients are diagnosed at early stage without nodal involvement, and for this subgroup, the standard treatment consists of surgery and postoperative radiotherapy, and systemic adjuvant therapy if necessary. Postoperative whole-breast irradiation serves as an adjuvant therapy following breast-conserving surgery that provides equivalent long-term survival comparable to radical mastectomy, and is now recognized as the standard treatment for early-stage breast cancer [2]. For node-negative patients, the hypofractionated schedule is commonly recommended as randomized trials have confirmed their safety and efficacy [3]. At our department, hypofractionated radiotherapy (42.56 Gy in 16 fractions) has been implemented for nearly all early-stage node-negative breast cancer patients in the recent 2 years.

Following the Quantitative Analysis of Normal Tissue Effects in the Clinic (QUANTEC) and Radiation Therapy Oncology Group (RTOG) consensus guidelines, the main organs at risk (OARs) for whole-breast radiotherapy include the heart, lung and contralateral breast [4, 5]. Whole-breast irradiation is generally performed with two opposed tangential beams to avoid the above-mentioned normal organs while simplifying the treatment plan. Tangential beam intensity-modulated radiation therapy (IMRT) may be employed to enhance the dose homogeneity [6–9]. Compared with other cancer sites, treatment planning for whole-breast irradiation is relatively simple yet contributes to a large proportion of the workload for the medical personnel in radiation oncology departments, and thus is an ideal candidate for automation. In the recent decade several published studies reported on automated treatment planning algorithms implementation for the head and neck, tangent breast, prostate and palliative spine [10–15]. These automated planning algorithms reduced the time and effort required to create personalized treatment plans, while also allowing the planning process to be highly standardized. The published studies generally showed similar target coverage and equivalent clinical acceptability of automated plans when compared with manual plans. For head and neck cancer planning, better OAR sparing favoring the automated plans were even reported [12].

We argue that a well-designed automated planning algorithm can improve current radiation oncology clinical workflow by standardizing plan quality, accelerating the treatment planning process, and improving employee productivity. We collected left-sided breast cancer patient manual treatment plans who received hypofractionated whole-breast irradiation as an adjuvant therapy for partial mastectomy at our hospital to prove this concept. These treatment plans were re-planned with an in-house-developed algorithm. The dosimetric quality was compared between the automated plans and manual plans.

## Methods

### Patient selection

A cohort of 99 patients diagnosed with left-sided stage I or stage II node-negative breast cancer who received postoperative whole-breast irradiation without nodal irradiation from year 2016 to 2018 at a tertiary center were included in this study. All patients were aged 20 years or older and received hypofractionated regimen of 42.56 Gy in 16 daily fractions.

### CT simulation and contouring

A customized immobilization device was used for all patients with the ipsilateral arm raised to maximize the precision and repeatability of the daily positioning for irradiation. Following patient immobilization, planning images were acquired with a computerized tomography (CT) scanner (SOMATOM Definition AS, Siemens, Germany) at a 3-mm slice thickness. Our department used the voluntary deep-inspiration breath-hold (DIBH) technique to reduce the cardiac radiation dose in breast cancer management. The patients performed a supervised breath hold during CT simulation and treatment.

For each patient in our study cohort, we re-planned with our auto-planning algorithm, based on previous approved contours. The treatment target and OARs were manually contoured based on the RTOG 1005 protocol guidelines [16]. The clinical target volume (CTV) included the whole left breast and was extended isotropically with a margin of 5 mm to form the planning target volume (PTV). The PTV was cropped to a distance of 4 mm from the patient’s skin surface. The contralateral breast, ipsilateral and contralateral lung, and heart were manually contoured as OARs.

### Manual treatment planning technique

**Fig. 1:**
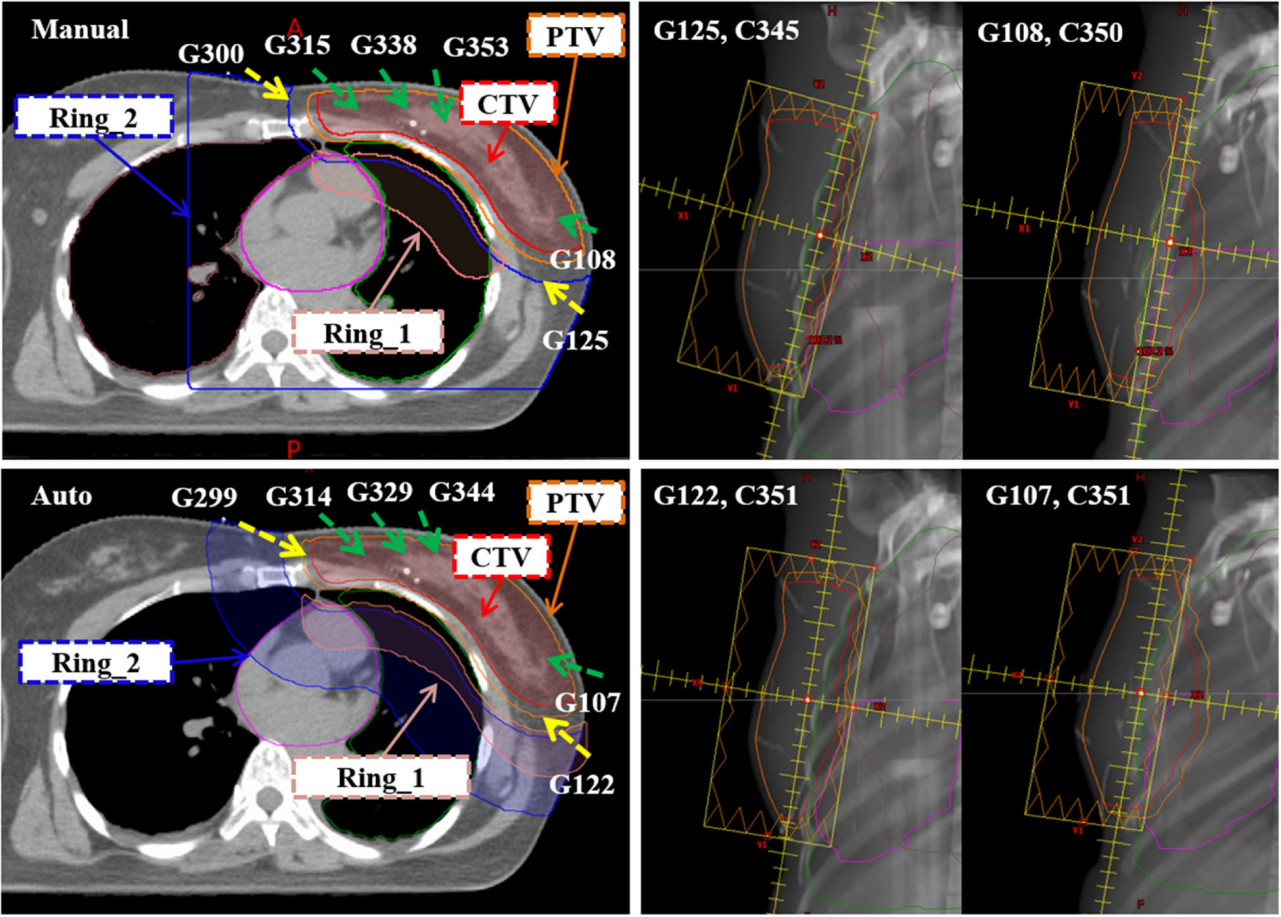
A representative CT axial view demonstrating beam arrangement and planning auxiliary structure (left panel) and beam’s-eye-view (right panel). Upper row: manual plan. Lower row: autoplan

All manual plans for hypofractionated whole-breast irradiation used six-field IMRT design with six MV photon beams at our department, created by experienced medical physicists. Two tangential beams (major fields) were manually assigned by the treatment planner to fit the PTV borders. Four oblique beams (minor fields) with reduced field size were subsequently created. All of the fields were extended at the breast apex to account for the breathing motion. The minor beam angles were adjusted to avoid the critical organs. Dose-volume constraints were set based on the RTOG 1005 protocol. Auxiliary structures Ring_1 and Ring_2 were manually created by the treatment planner. The areas of relative overdose (hotspots) were eliminated by assigning the hot spots region, isodose area of V_105%_, as a constraint structure to the objective template. The elimination of hotspots usually needs to be repeated several times. A beam’s-eye-view example of the manual plan is shown in Fig. 1.

### Automatic treatment plan (autoplan) generation

**Fig. 2:**
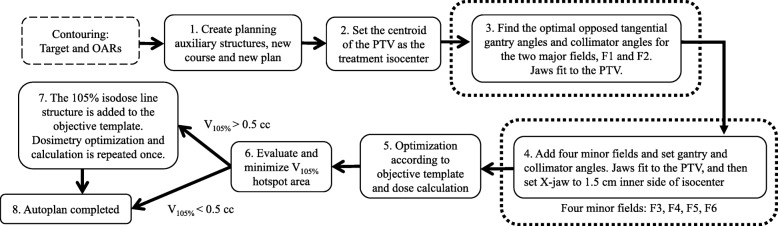
The schematic diagram of an autoplan. Detailed description is in the Methods and Materials section

The automated plan-generating computer algorithm is an in-house-developed C# program created as a script of the Varian Eclipse Scripting Application Programming Interface (ESAPI). The generated autoplan schematic diagram is shown in Fig. 2. The detailed process is described as follows: (1) Automatically create auxiliary structures Ring_1 and Ring_2 by extending the PTV posteriorly (Fig. 1), and then create a new plan under a new course for a selected patient. (2) Set the centroid of the PTV as the treatment isocenter. (3) Identify the optimal opposed tangential gantry angles and collimator angles by iterating the angles one degree at a time. For the gantry angle, iterate between 270 and 330 degrees (300 ± 30 degrees range) for major field F1, and between 90 and 150 degrees (120 ± 30 degrees range) for major field F2. For the collimator angle, iterate between 330 and 30 degrees (0 ± 30 degrees range). For each iteration, the jaw fits to the PTV and then the field area (defined by the X and Y jaws) is calculated. We choose the gantry and collimator angles pair which has the smallest field area. (4) Add four minor fields F3, F4, F5 and F6. The three minor fields F3, F4 and F5 are set based on F1. The gantry angles are set as follows: F3 = F1 + 15 degrees, F4 = F1 + 30 degrees, F5 = F1 + 45 degrees, and the collimator angles of these three subfields are set as F1. The fourth minor field F6 is determined based on F2, with F6 gantry angle = F2–15 degrees, and F6 collimator angle is set as F2. For the minor fields, the jaws fit to the PTV, and then the inner side of the jaw opens 1.5 cm away from the isocenter (Fig. 1). (5) Optimize plan according to the objective template (Additional file 1: Table S1) and calculate dose. (6) Evaluate and minimize V_105%_ hotspot area. If V_105%_ < 0.5 cc, the plan is completed (Step 8). If V_105%_ > 0.5 cc, the constraint structure with region of 105% isodose area will be automatically created, and the program moves to the next step (Step 7). (7) The V_105%_ constraint structure is added to the objective template (Additional file 1: Table S1). Plan optimization and dose calculation are repeated once again. (8) The autoplan is completed.

All automated plans were generated using the Eclipse treatment planning system (TPS) under a research license (Version 15.6, Varian Medical Systems Inc., Palo Alto, California, USA). Analytical Anisotropic Algorithm was used for dose calculation for all plans. All automated plans were simulated on the TPS but not delivered clinically.

### Plan evaluation and analysis

The DVHs and dosimetric parameters of all manual plans and autoplans were collected and calculated for data analysis. The mean DVH band was calculated and reflected the volume-dose distribution with 95% confidence interval. For plan evaluation, the following parameters were recorded. The V_110%_ of the prescribed dose inside the body was analyzed for hotspot evaluation. For PTV coverage evaluation, V_95%_ was collected. The dose homogeneity index (HI) of PTV was measured by D_5%_ divided by D_95%_ (D_5%_ / D_95%_) [17]. The conformity index (CI) of PTV was defined as BV_95%_ / PTV (BV_95%_ = the volume of the body receiving 95% of the prescribed dose) [18]. For dose analysis of the OARs, the V_16Gy_ and D_mean_ were reported for the ipsilateral lung, V_20Gy_ and D_mean_ for the heart, and V_5Gy_ and D_max_ for the contralateral breast per RTOG 1005 protocol. The monitor unit (MU) was calculated by summing the MUs of all fields in a treatment plan.

### Statistical analysis

The dosimetric parameters and total MUs were evaluated by paired two-tailed *t*-test for the manual and automatic treatment plans. One-way analysis of variance (ANOVA) was applied to evaluate the differences between OAR parameters of patients with different breast sizes stratified into three groups (small, medium and large) according to the CTV with a cutoff of 300 ml and 600 ml. A *p*-value less than 0.05 was considered statistically significant. Data are presented as mean value ± standard deviation (SD) if not otherwise specified. All statistical analyses and graphs were performed using the statistical software R version 3.5.2.

## Results

The mean difference between the gantry and collimator angles for F1 and F2 fields between autoplans and manual plans were 2.69 ± 3.00, 5.24 ± 5.63, 2.26 ± 2.67 and 2.42 ± 2.81 (Mean ± SD) degrees, respectively. For hotspot V_105%_ evaluation of all 99 autoplans, 66 (67.7%) plans had smaller V_105%_ after the automatic optimization step (Step 6), 24 (24.2%) did not have V_105%_ > 0.5 cc, and eight (8.1%) plans still had V_105%_ > 0.5 cc after optimization.

Table 1 listed the dosimetric parameters for the hotspot area (V_110%_), the PTV coverage, ipsilateral lung, heart and contralateral breast. There was no significant difference in hotspot volume between the two approaches. However, compared with the manual plan, the autoplan performed slightly better in ipsilateral-lung-sparing (in terms of V_16Gy_, 14.0 ± 3.6 vs. 13.3 ± 3.0, *p* < 0.01, and mean dose, 6.6 ± 1.5 vs.6.3 ± 1.2, *p* < 0.01), but was inferior in contralateral-breast-sparing (in terms of maximal dose, 3.5 ± 7.3 vs.5.0 ± 8.7, *p* < 0.01). Additionally, the autoplan was generally superior to the manual plan in CI (1.24 ± 0.06 vs. 1.30 ± 0.09, *p* < 0.01) and MU (1010 ± 46 vs. 1205 ± 187, *p* < 0.01).

**Table 1:** The mean, minimal and maximal value of the parameters for evaluation of PTV coverage and OARs constraints of all plans. Data analysis for comparison between manual plans and autoplans was done with paired two-tailed *t*-test. A *p*-value less than 0.05 was considered statistically significant

	Manual	Auto	
Parameter	Mean value ± SD [Min, Max]	Mean value ± SD [Min, Max]	*p*-value
Body			
V_110%_[%]	0.07 ± 0.29 [0.0, 2.15]	0.03 ± 0.23 [0.0, 2.15]	0.29
PTV			
V_95%_[%]	98.5 ± 0.5 [96.7, 99.5]	98.5 ± 0.6 [96.4, 99.9]	0.40
Ipsilateral lung			
V_16Gy_[%]	14.0 ± 3.6 [5.1, 28.3]	13.3 ± 3.0 [4.3, 26.9]	< 0.01
Mean[Gy]	6.6 ± 1.5 [3.0, 12.7]	6.3 ± 1.2 [2.7, 10.9]	< 0.01
Heart			
V_20Gy_[%]	0.90 ± 1.30 [0.0, 8.0]	0.90 ± 1.10 [0.0, 4.4]	0.79
Mean[Gy]	1.4 ± 0.6 [0.5, 4.0]	1.4 ± 0.6 [0.6, 3.3]	0.30
Contralateral breast			
V_5Gy_[%]	0.22 ± 1.29 [0.0, 10.40]	0.46 ± 2.50 [0.0, 22.83]	0.31
Max[Gy]	3.5 ± 7.3 [0.2, 43.8]	5.0 ± 8.7 [0.2, 44.6]	< 0.01
CI	1.30 ± 0.09 [1.10, 1.63]	1.24 ± 0.06 [1.14, 1.41]	< 0.01
HI	1.08 ± 0.02 [1.05, 1.13]	1.07 ± 0.02 [1.05, 1.13]	< 0.01
MU	1205 ± 187 [775, 1650]	1010 ± 46 [772, 1353]	< 0.01

**Fig. 3:**
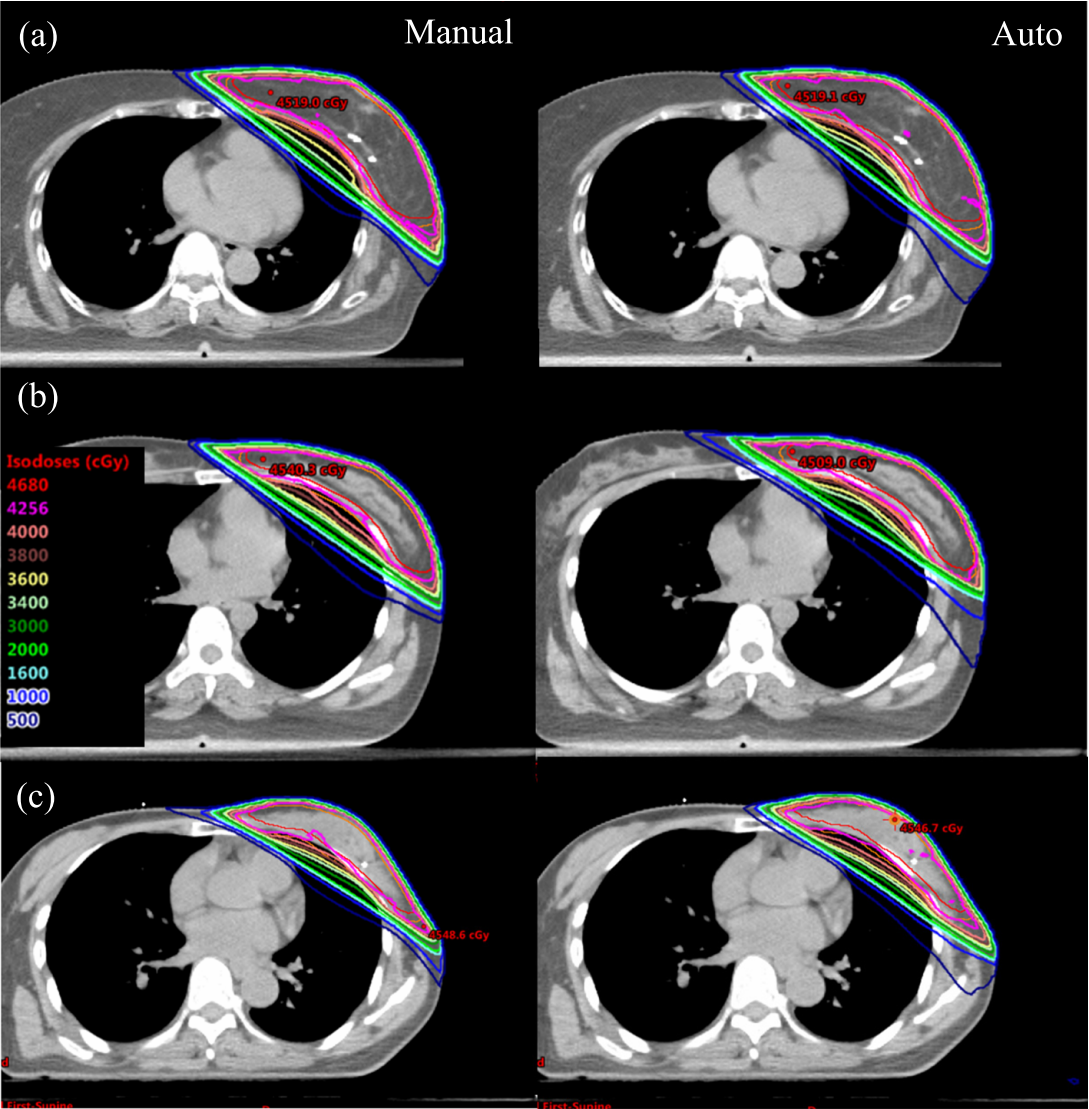
Dose distributions in three representative patients with different breast sizes. Row (**a**) represents large breast size, row (**b**) medium breast size, and row (**c**) small breast size. Left column shows the dose distributions of autoplan, and right column the manual plan. All plans prescribed 42.56 Gy in 16 fractions. Isodose lines were drawn with different colors

The average time to generate an autoplan was only five to 6 min irrespective of each patient’s anatomical variance, while the manual planning time ranged from 1 h to one and a half hours. Figure 3 showed the autoplan and manual plan dose distributions for patients with different breast sizes (large, medium and small). Table 2 shows the ipsilateral lung, contralateral breast and heart dose parameters for all plans stratified by the patients’ breast sizes. In general, different breast sizes did not have much impact on the autoplan parameters, but for manual plans, there was slightly better ipsilateral-lung-sparing in smaller breast (V_4Gy_ = 25.7 ± 3.7%) than in larger (V_4Gy_ = 28.1 ± 6.9%) or medium-sized (V_4Gy_ = 29.7 ± 5.8%) breast (*p* < 0.01).

**Table 2:** The constraints evaluation of ipsilateral lung, contralateral breast and heart of all plans stratified by different breast sizes. *L* large breast size (*n* = 18), *M* medium breast size (*n* = 52), *S* small breast size (*n* = 29)

	Manual (Mean value ± SD)				Auto (Mean value ± SD)			
Parameter	L	M	S	*p-value*	L	M	S	*p-value*
Ipsilateral lung								
V_16Gy_[%]	12.9 ± 3.8	14.5 ± 3.6	13.7 ± 3.5	0.25	13.0 ± 3.5	13.5 ± 3.3	13.1 ± 2.2	0.78
V_4Gy_[%]	28.1 ± 6.9	29.7 ± 5.8	25.7 ± 3.7	< 0.01	28.8 ± 7.0	28.4 ± 5.2	26.8 ± 2.4	0.30
Contralateral breast								
V_5Gy_[%]	0.0 ± 0.0	0.3 ± 1.7	0.0 ± 0.4	0.46	0.0 ± 0.0	0.8 ± 3.4	0.0 ± 0.0	0.23
Max[Gy]	2.0 ± 2.3	4.7 ± 9.6	2.2 ± 2.6	0.19	3.6 ± 4.4	6.6 ± 11.3	2.8 ± 2.9	0.13
Heart								
V_20Gy_[%]	0.9 ± 1.4	0.9 ± 1.3	0.9 ± 1.2	0.98	1.2 ± 1.5	0.9 ± 0.9	0.9 ± 1.1	0.42
Mean[Gy]	1.5 ± 0.6	1.4 ± 0.6	1.3 ± 0.7	0.37	1.7 ± 0.7	1.4 ± 0.5	1.2 ± 0.5	0.03

**Fig. 4:**
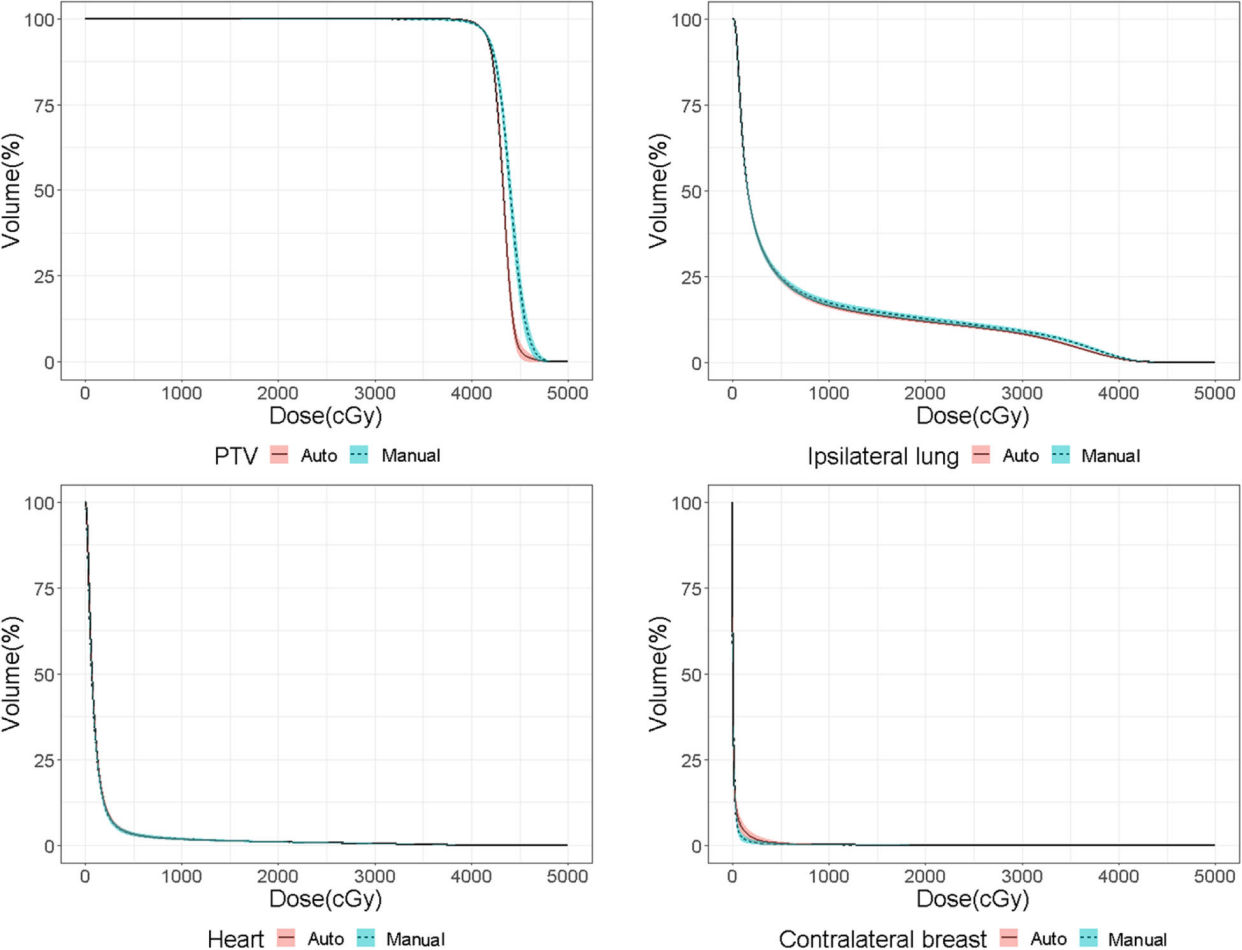
Mean DVH curves with 95% confidence interval (shaded area) for the PTV, ipsilateral lung, heart and contralateral breast for autoplans (solid red lines) and manual plans (dashed green lines)

For comparison purposes, the V_95%_ of the PTV for the manual plan was normalized to the V_95%_ of the PTV for the autoplan for each patient. As shown in Fig. 4, the DVH curves of autoplans and manual plans for the heart nearly overlapped. However, compared with the manual plans, the DVH curve of autoplans had a better ipsilateral-lung-sparing trend but less contralateral-breast-sparing.

## Discussion

Manual treatment planning is an iteratively trial-and-error process that involves repeatedly adjusting beam angles and objective template parameters based on each treatment planner’s experience, skill and knowledge. This manual approach results in high workload and potentially suboptimal plan quality due to the operator-dependent preferences and priorities of the treatment planner and the physician. This study demonstrates that a well-designed automated treatment plan-generating algorithm can reduce treatment plan variability and standardize treatment plans. This approach results from more ideal, standardized gantry angle and collimator angle design with less inter-planner variability in autoplans. As it is never an easy task to convince medical personnel to embrace a new technology or automate clinical workflow, we designed this study to compare the manual plans and in-house-developed autoplans to examine the quality and feasibility of the automated plan-generating program for this large cohort study.

The auto-planning algorithm for whole-breast irradiation emulated the manual plan generation process, and required only five to 6 min to generate an optimized plan. Compared with the available published literature for automated breast irradiation planning, the auto-planning algorithm developed in this study saved time in terms of plan generation and optimization. A study published in 2010 used rapid but robust two-field tangential IMRT planning with a speed of 9 min per plan for 53 patients with the purpose of selecting patients that might benefit more from the deep inspiration breath hold technique [19]. A Netherland’s study presented a single-click optimized autoplan software with planning time of 20 min for breast and prostate and 7 min for palliative vertebrae [14]. A Canadian study conducted by Purdie et al. used the Pinnacle treatment planning system to generate optimized whole-breast irradiation treatment plan using radio-opaque markers placed during CT simulation as the only input, with the mean time of 7 min per plan generation [10, 11].

Aside from the timesaving advantage of automated treatment planning, the above-mentioned studies generally showed equivalent autoplan clinical acceptability to manual plans with equivalent target dose and OAR constraint parameters, as well as similar treatment delivery time, or MUs. The advantage of applying the autoplans in this study include the following. First, the machine workload is minimized as the autoplans use fewer MUs to achieve similar PTV coverage and OAR sparing comparable with the manual plans overall. Other previously mentioned published studies used two-field or four-field design, with no difference in MUs between automated plans and manual plans. Second, there is a slightly higher homogeneity in PTV dose coverage for autoplans. The DVH analyses (Fig. 4) demonstrate a steeper slope in the average DVH curve for the PTV of the autoplans compared with the manual plans. Third, the OAR dose parameters and target coverage are uniform and consistent in autoplans than in manual plans. The narrow 95% confidence interval shown in the DVH analyses of the 99 autoplans indicates the uniform consistency of the dose parameters.

The automated planning algorithm in this study is highly efficient as it uses six-field IMRT and could generate an optimized plan with high conformity and homogeneity in five to 6 min on a standalone workstation. The observed advantage of autoplans implies that when applying a more complicated field design, an automated planning algorithm may not only save much time for medical physicists and dosimetrists, but also find acceptable or even better solutions for a standardized and optimized treatment plan than a human could within a limited time. Four to five hypofractionated breast cancer treatments are planned per week at our oncology department on average. The estimated working hours saved per month is therefore 18 h per month. Moreover, a multi-field complex planning task requires great experience, many calculations and much trial-and-error, which may be overwhelming in difficult cases. Our automated planning algorithm achieves the best gantry angles, collimator angles, MUs for each field and minimal hotspots through iteration and optimization. This would be a daunting task if reproduced manually. Applying an automated planning algorithm can also reduce inter-planner variability between experienced and non-experienced treatment planners, which may contribute to plan quality control and educational training.

It is also interesting to note that, as shown in the DVH curves in Fig. 4 and parameters in Table 1, the autoplanning algorithm tends to maximize the PTV coverage without compromising it in order to spare the lung dose, which probably results from the strict adherence to the planning algorithm design (Fig. 1) and the objective template (Additional file 1: Table S1). In reality, human doctors and physicists might accept a trade-off between the PTV coverage, lung dose and other OAR constraints, considering a patient’s medical or clinical condition, such as chronic lung disease or impaired heart function. As the criterion for plan approval is according to each clinician’s discretion, our autoplanning algorithm has the advantage of quickly generating several optimized plans using different objective templates, allowing clinicians to choose the ideal one to approve for treatment delivery based on clinical judgement. However, physicist manual refinement of automatically generated plan may be required based on individual patient’s condition.

To date, automated workflow has been developed and implemented in various businesses but the pace of applying automation into health care is not as fast as expected. In the recent two decades, the rapid progress in computerized radiotherapy treatment planning systems has led to great interest in the possibility of automating radiotherapy workflow, which includes automated target delineation (auto-segmentation), automated treatment planning, automated real-time adaptive radiotherapy and automated quality assurance [20–25]. This study presents a well-designed fully automated planning algorithm for standardized whole-breast radiotherapy treatment plan generation with a large cohort of 99 patients. By retrospectively analyzing and comparing the autoplans and manual plans, we prove that this algorithm has great potential in clinical applicability, and may improve radiotherapy treatment planning workflow in efficiency and standardization. While the autoplanning algorithm may save much time and reduce the repetitive planning workload of medical physicists and dosimetrists if implemented clinically, it may also help medical personnel focus on tasks requiring innovation and creativity. In the future, we seek to develop a program that automatically extracts clinical information and combine it with the automated planning algorithm to develop a personalized automated treatment planning program, thereby increasing its applicability.

## Conclusions

An automated treatment-planning algorithm for standardized and optimized hypofractionated whole-breast irradiation plan generation is developed to reduce the radiation oncology personnel workload and improve human performance and productivity. Autoplans with higher efficacy (more MU-saving) and with more uniformity and consistency in plan parameters are generated by the algorithm when compared with manual plans. We conclude that a well-designed automated treatment planning program can improve current clinical practice in the radiation oncology field, and argue that automatic treatment planning will become part of the standard workflow in radiation oncology departments at medical centers.

## Supplementary information

**Additional file 1 : Table S1.** The objective template for dosimetry optimization.

## Data Availability

The autoplanning code during the current study are available from CY Lin (cylin1230@gmail.com) to any reader directly on reasonable request.

## Abbreviations

CI: Conformity index
CT: Computerized tomography
CTV: Clinical target volume
DIBH: Deep-inspiration breath-hold
DVH: Dose-volume histogram
ESAPI: Eclipse scripting application programming interface
HI: Homogeneity index
IMRT: Intensity-modulated radiation therapy
MU: Monitor unit
OAR: Organ at risk
PTV: Planning target volume
QUANTEC: Quantitative analysis of normal tissue effects in the clinic
RTOG: Radiation therapy oncology group
SD: Standard deviation
TPS: Treatment planning system
