# Supplementary material for: Automated Hypofractionated IMRT treatment planning for early-stage breast Cancer
Source: Radiat Oncol. 2020 Mar 17;15:67. doi: 10.1186/s13014-020-1468-9 (PMC7077022; doi:10.1186/s13014-020-1468-9)
Supplement: Supplementary file 1 — Additional file 1 : Table S1. The objective template for dosimetry optimization. [file 13014_2020_1468_MOESM1_ESM.docx]

**Supplementary Table.** The objective template for dosimetry optimization.

| **Structure** | **Type** | **Volume[%]** | **Dose[cGy](%D_p_)** | **Priority** |
| --- | --- | --- | --- | --- |
| **CTV** | Upper | 0 | 4476 (105%) | 200 |
|  | Lower | 100 | 4350 (102%) | 200 |
| **PTV** | Upper | 0 | 4476 | 275 |
|  | Lower | 100 | 4306 | 175 |
| **Body** | Upper | 0 | 4476 | 350 |
| **Contralateral breast** | Upper | 0 | 250 | 125 |
|  | Upper | 5 | 100 | 125 |
| **Lung** | Upper | 4 | 3500 | 100 |
|  | Upper | 5 | 1900 | 100 |
|  | Upper | 8 | 500 | 100 |
| **Heart** | Upper | 2 | 600 | 100 |
|  | Upper | 4 | 300 | 100 |
|  | Upper | 12 | 175 | 100 |
| **Ipsilateral lung** | Upper | 12 | 1800 | 120 |
| **Ring_1** | Upper | 0 | 4050 | 100 |
|  | Upper | 0 | 3800 | 100 |
|  | Upper | 5 | 3600 | 100 |
| **Ring_2** | Upper | 0 | 4350 | 250 |
| **V_105%_** | Upper | 0 | 4468 | 150 |

V_105%_ = The volume of Body receiving 105% of the prescribed dose
